# Transcriptome Analysis Revealed the Molecular Response Mechanism of High-Resistant and Low-Resistant Alfalfa Varieties to Verticillium Wilt

**DOI:** 10.3389/fpls.2022.931001

**Published:** 2022-06-16

**Authors:** Fang Li, Xi Chen, Bo Yang, Yingjie Guang, Dandan Wu, Zunji Shi, Yanzhong Li

**Affiliations:** State Key Laboratory of Grassland Agro-Ecosystems, Center for Grassland Microbiome, Gansu Tech Innovation Center of Western China Grassland Industry, College of Pastoral Agriculture Science and Technology, Lanzhou University, Lanzhou, China

**Keywords:** alfalfa, Verticillium wilt, *Verticillium alfalfae*, resistance mechanism, transcriptomics

## Abstract

Following infestation by Verticillium wilt, alfalfa (*Medicago sativa* L.) often shows symptoms such as disease spots, leaf loss, stem, and leaf yellowing, resulting in the decline of alfalfa yield and quality and causing significant losses to the alfalfa industry. The popularization and planting of disease-resistant varieties is the most effective method to prevent and control Verticillium wilt of alfalfa. Therefore, it is particularly important to reveal the resistance mechanism of Verticillium wilt resistant varieties of alfalfa. In this study, the physiological and biochemical indexes were measured on days 7, 14, 21, and 28 after inoculation with *Verticillium alfalfae* for investigating the response mechanisms of two alfalfa varieties, high-resistant WL343HQ, and low-resistant Dryland. Transcriptome sequencing of alfalfa samples infected with *V. alfalfae* and uninfected alfalfa samples was performed to analyze the potential functions and signaling pathways of differentially expressed genes (DEGs) by GO classification and KEGG enrichment analysis. Meanwhile, weighted gene co-correlation network analysis (WGCNA) algorithm was used to construct a co-expression network of DEGs. Inoculation with *V. alfalfae* significantly affected net photosynthetic rate, stomatal conductance, chlorophyll content, MDA content, JA and SA concentrations, and NO and H_2_O_2_ contents in both WL343HQ and Dryland inoculated with *V. alfalfae*. Most of the transcription factors in plants were classified in the WRKY, NAC, and bHLH families. WGCNA analysis showed that the number of transcription factors related to plant growth and disease resistance was higher in the corresponding modules of WL343HQ disease groups on days 7 and 28 (WVa) and (WVd) than in the corresponding modules of Dryland disease groups on days 7 and 21 (HVa) and (HVc). These findings provide data for further gene function validation and also provide a reference for in-depth studies on interactions between plants and pathogens.

## Introduction

Alfalfa (*Medicago sativa* L.) is widely grown in the United States, Canada, China, Argentina and other countries. With the expansion of alfalfa cultivation, diseases have become one of the main limitations to the production and utilization of alfalfa. Among them, the most common alfalfa diseases are caused by fungi. A 35 alfalfa diseases caused by more than 70 pathogenic fungi have been reported all over the world (Samac et al., 2014; Wen et al., 2015). Verticillium wilt caused by *Verticillium alfalfae* is a devastating disease in alfalfa production (Peaden et al., 1985). The disease was first reported in Sweden in 1918, thereafter it spread widely in North America (Grau et al., 1981; Gordon et al., 1989). In 2014, Verticillium wilt was detected in Minle County, Gansu Province, China, and the average incidence rate of the disease in Minle was 45%, with 100% isolation of the pathogen in stem and root of symptomatic plants (Xu et al., 2016).

The invasion of *V. alfalfae* causes a series of physiological and biochemical responses, including the increase of membrane lipid peroxidation products and the decrease of soluble sugar content and nutrient uptake (Gharbi et al., 2016; Bibi et al., 2017). Furthermore, *Verticillium dahliae* is able to cause disease by inducing the expression of related genes and regulating signaling pathways. *GhWRKY70D13* gene was upregulated after inoculation with *V. dahliae*, and knockdown of this gene improved resistance to *V. dahliae* in both resistant and susceptible plants (Xiong et al., 2020). Differentially expressed genes (DEGs) in olive samples of susceptible (Picual) and resistant (Frantoio) varieties were analyzed by transcriptomic techniques after 2 weeks of inoculation with *V. dahliae*, and it was found that the amount of mRNA of *V. dahliae* in susceptible varieties was significantly higher than that in resistant varieties (Jimenez-Ruiz et al., 2019). The transcriptome results of Verticillium wilt of eggplants revealed that DEGs associated with Verticillium wilt were mainly involved in “amino acid transport and metabolism,” “cytoskeleton” and “cellular activity” (Yang et al., 2019). Compared to control plants, 111 common DEGs were identified in diseased plants and most of them were enriched in “signal transduction pathway.”

The popularization and planting of disease-resistant varieties is the most effective method to prevent and control Verticillium wilt of alfalfa. Therefore, it is particularly important to reveal the resistance mechanism of Verticillium wilt resistant varieties of alfalfa. After infected by *Verticillium alfalfae*, plants produced disease resistance-related enzymes such as phenylpropanoid metabolic enzymes, participating in lignin and salicylic acid synthesis for reinforcing cell wall and building systemic resistance (MauchMani and Slusarenko, 1996; Smit and Dubery, 1997). Signal transduction molecules such as hydrogen peroxide (H_2_O_2_), salicylic acid (SA), and jasmonic acid (JA) induced resistance responses synergistically or individually (Yao et al., 2011; Dhar et al., 2020). H_2_O_2_ can effectively resist the invasion of pathogenic bacteria and the expansion of pathogenic mycelium by triggering a series of defense responses such as allergic response and programmed cell death (Dat et al., 2000; Mellersh et al., 2002). SA can improve plant tolerance to *V. alfalfae* (Zhen and Li, 2004). JA was associated with plant resistance, and tomato plants lacking JA were more susceptible to *V. alfalfae* (Thaler et al., 2004; Tjamos et al., 2005). After infected by Verticillium wilt, regulatory genes that induce specific defense responses were upregulated in cells near the point of infection (Hill et al., 1999), for example, *Ve* gene can improve resistance of tomatoes to *V. dahliae* (Nachmias et al., 1987).

In this study, physiological and biochemical indexes and transcriptome sequencing were performed after inoculation with *V. alfalfae* for investigating the resistance mechanism of different alfalfa varieties. These findings provide new insights for in-depth studies on the interactions between plants and pathogenic bacteria.

## Materials and Methods

### Sample Collection

We selected a high resistance variety (WL343HQ) and a low resistance variety (Dryland) for this study. The alfalfa seeds were sterilized and placed on sterilized filter paper of culture dish for germination. The soil used for the experiments was turfy soil, which was collected from a grassland in the same place. Impurities in the soil samples, such as gravel, leaves, and grass roots, were removed during the soil collection. The soil was sterilized by γ-radiation and sealed. Each pot contained 1.0 kg. The pathogenic fungus used in this experiment was the *V. alfalfae* strain LYZ0257 isolated from Minle County. A spore suspension of 1 × 10^6^/ml was prepared with sterile water. Two alfalfa varieties, WL343HQ and Dryland, were divided into disease groups (WL + V and HD + V) and control groups (WL and HD). After 3 days of germination, the well-grown seedlings were transplanted into pots with sterilized soil. Each treatment had 10 pots, 40 pots in total, nine alfalfa plants per pot. The plants were watered every 3 days and maintained in the inoculation room, with a 12 h photoperiod, 22 and 18°C day and night temperatures, and 65–90% relative humidity. Alfalfa was inoculated with *V. alfalfae* after 6 weeks of growth by spray method. The inoculation dose was 10 ml of spore suspension of *V. alfalfae* per pot. The control groups were sprayed with 10 ml of sterile water per pot. The leaves were taken from the same parts of alfalfa on days 7, 14, 21, and 28, respectively, then frozen in liquid nitrogen and stored at −80°C for the determination of physiological and biochemical indicators and transcriptome sequencing. The transcriptome sequencing had three replicates for each treatment.

### Determination of Physiological and Biochemical Indicators

The number of diseased plants per pot was counted separately and the incidence rate of disease was the mean of 10 replicates. Disease index, representing both incidence rate and symptom severity of disease, can be calculated as Disease index = disease ratio×disease severity×100%. Small segments were cut from the thickest stalk of each plant, and the stalks were sterilized in 75% alcohol and 1% NaClO for 1 min, respectively, and then rinsed 3–4 times with sterile water. After absorbing the surface water of the stalks with sterilized filter paper, the stalks were cut into small sections of about 2 mm and placed on PDA medium. The plates were placed in the incubator at 23–25°C and observed daily. The plant carrier rate of *V. alfalfae* was counted on the 4th day. Leaf net photosynthetic rate and stomatal conductance were measured using a GFS-3000 photosynthesizer (Heinz Walz GmbH Co., Ltd., Effeltrich, Germany) from 9: 00 a.m. to 12: 00 p.m. on days 7, 14, 21, and 28, respectively. The chlorophyll content of the plants was determined by the acetone extraction method (Fang et al., 2020). The measurement was performed using ELISA kits of SA and JA (Nanjing Jiancheng Biological Engineering Research Institute Co., Ltd., Nanjing, China). The absorbance value of each well was measured sequentially at 450 nm using an RT-6100 Microplate Reader (Rayto Life and Analytical Sciences Co., Ltd., Shenzhen, China). The ELISA kits for NO and H_2_O_2_ (Nanjing Jiancheng Biological Engineering Research Institute Co., Ltd., Nanjing, China) were used to detect the amount of NO and H_2_O_2_ in the samples, and the absorbance values were measured at 550 nm and 405 nm, respectively. The superoxide dismutase (SOD) and peroxidase (POD) activities were measured by the nitrogen blue tetrazolium and guaiacol methods (Shi et al., 2014). The thiobarbituric acid method was used to determine the MDA concentration in plants (Yin et al., 2019).

### Transcriptome Sequencing

The leaves from the same parts of WL343HQ and Dryland were collected and sent to Biomarker Technologies Co., Ltd. (Beijing, China) for transcriptome sequencing. Samples were selected on days 7, 14, 21, and 28 for WL343HQ disease groups (WVa, WVb, WVc, and WVd), WL343HQ control groups (Wa, Wb, Wc, and Wd), Dryland disease groups (HVa, HVb, HVc, and HVd), and Dryland control groups (Ha, Hb, Hc, and Hd). Three biological replicates were conducted per group, with a total of 48 samples. RNA extraction from alfalfa leaves was performed using the Tiangen DP441 kit (Tiangen Biotech Co., Ltd., Beijing, China). The obtained RNA extracts were measured for concentration and purity using Nanodrop 2000 (Thermo Fisher Scientific Co., Ltd., Waltham, MA, United States). Agilent 2,100 Bioanalyzer (Agilent Technologies, Inc., Santa Clara, CA, United States) and LabChip GX Nucleic Acid Analyzer (PerkinElmer Instrument Co., Ltd., Shanghai, China) were used to check the integrity testing. Samples with a 260:280 ratio of ≥2.0 and RNA integrity number of ≥8 were subjected to transcriptome sequencing. After the samples were tested, cDNA libraries were constructed and sequenced using the Illumina platform. Fastq format raw data were processed through in-house Perl scripts for quality control. Clean data were obtained by removing adapter containing reads and low-quality raw data reads. At the same time, the Q30, GC-content, and sequence duplication level of the clean reads were calculated. All downstream analyses were performed on high-quality and clean data.

### Quantitative Real-Time PCR Analysis

To validate the results of transcriptome analysis, the expression levels of 12 DEGs were detected by quantitative real-time PCR (qPCR) with three independent biological replicates for each sample. The primers were designed using Primer Designer 3.0 software, and 18S ribosomal RNA was chosen as the internal reference (Wang et al., 2015). The primers used in this study were listed in Supplementary Table 1. A 1 μg RNA was used to synthesize cDNA by Takara PrimeScript RT reagent Kit (Takara Biomedical Technology Co., Ltd., Beijing, China). qPCR assays were performed on CFX96 Touch Real-Time PCR Detection System (Bio-Rad Laboratories Co., Ltd., Hercules, CA, United States). The qPCR reaction conditions were set: 95°C, 3 min; 95°C for 3 s, 60°C for 30 s, 40 cycles. All treatments were repeated three times technically to reduce experimental errors due to handling and the instrument. Finally, the qPCR data were analyzed based on melting curve analysis using the ΔΔCT method (Livak and Schmittgen, 2001) and the relative gene expression was measured and normalized compared to control expression levels, and the control group was set to 1 for comparison of different groups.

### Statistical Analysis

The physiological and biochemical indexes were entered into Microsoft Excel 2020 for collation and calculation. Homogeneity of variance was detected by Levene test in SPSS 20.0 statistical software. Significant differences between treatments were analyzed by Tukey’s method (*p* < 0.05). The data of qPCR validation results of the genes were entered into Microsoft Excel 2020 and plotted after calculation by the ΔΔCT method (Livak and Schmittgen, 2001). The analysis of transcriptome sequencing data was mainly performed through Biomarker Cloud platform (Biomarker Technologies Co., Ltd., Beijing, China). The DESeq R package (v1.10.1) was used to perform differential expression analysis between different groups. Fragments per kilobase of transcript per million mapped reads (FPKM) were used to verify the transcriptional expression levels of the samples. The co-expression network was analyzed by weighted gene co-correlation network analysis (WGCNA) and mapped by R 4.1.0 software to describe the modules among genes. In addition, the core gene network was constructed and plotted by Cytoscape v.3.4.0 software.

## Results

### Physiological and Biochemical Indicators

**Figure 1:**
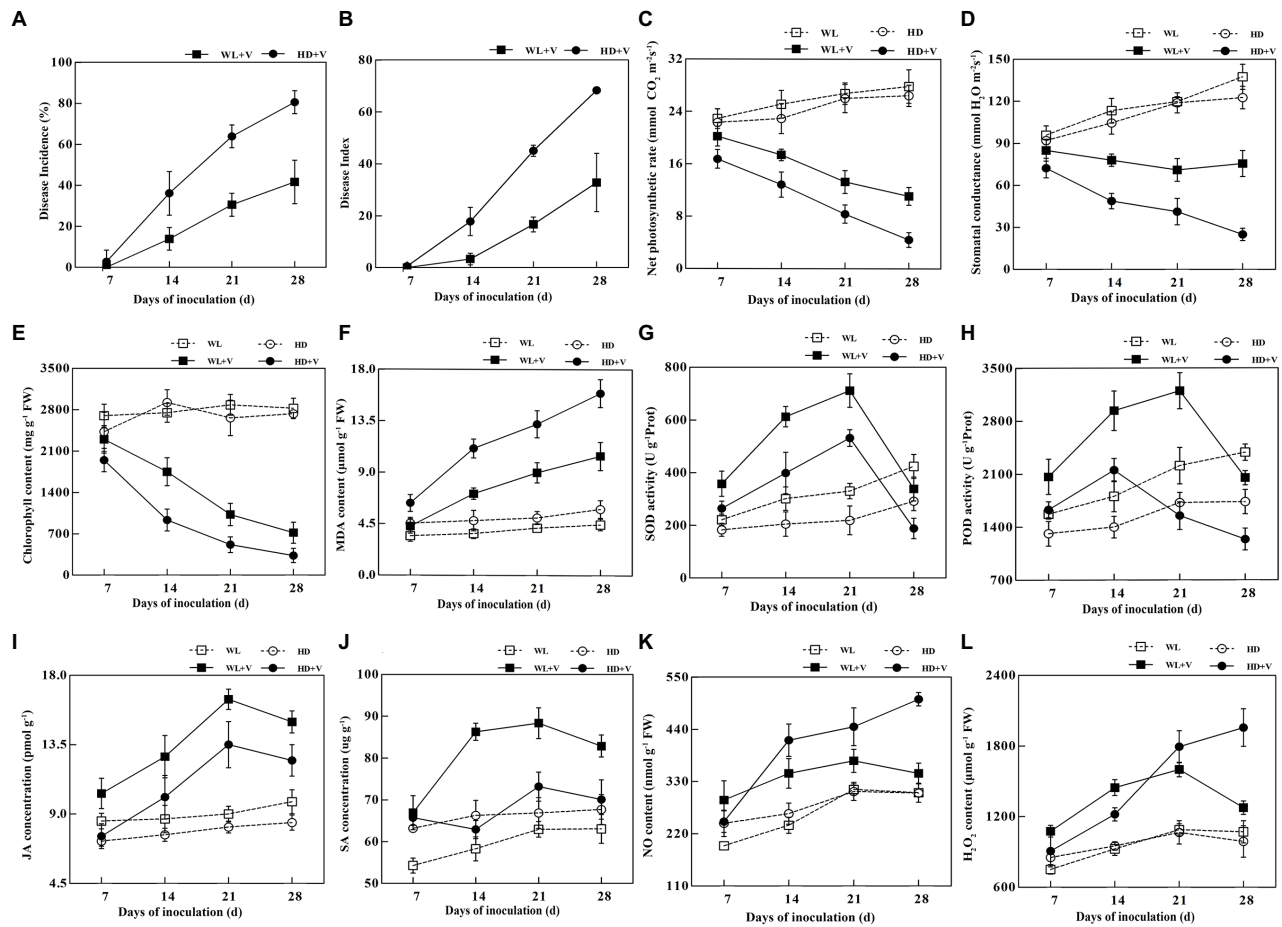
Linear charts of physiological and biochemical indicators of WL343HQ inoculated with *Verticillium alfalfae* (WL + V), Dryland inoculated with *V. alfalfae* (HD + V), and control groups (WL and HD). **(A)** Disease incidence. **(B)** Disease index. **(C)** Net photosynthetic rate. **(D)** Stomatal conductance. **(E)** Chlorophyll content. **(F)** MDA content. **(G)** SOD activity. **(H)** POD activity. **(I)** JA concentration. **(J)** SA concentration. **(K)** NO content. **(L)** H_2_O_2_ content. Data are presented as mean ± SD; *n* = 10 for each treatment.

After inoculation with *Verticillium alfalfae*, typical symptoms of verticillium wilt were seen in WL343HQ inoculated with *V. alfalfae* (WL + V) and Dryland inoculated with *V. alfalfae* (HD + V), and *V. alfalfae* was isolated from the plant stalks of both varieties. On day 7, symptoms of Verticillium wilt were found on the Dryland, which had a 2.78% infection rate, while no obvious symptoms were seen on the WL343HQ. On days 14, 21, and 28, the disease incidence rate of Dryland was 36.11, 63.89, and 80.55%, and the disease incidence rate of WL343HQ was 13.89, 30.55, and 41.66%, respectively (Figure 1A). On days 14, 21, and 28, the disease indices of Dryland were 17.78, 45 and 68.33, and the disease indices of WL343HQ were 3.33, 16.67, and 32.78, respectively (Figure 1B). There was no significant difference in disease incidence rate and indices between WL343HQ and Dryland on day 7. On days 14, 21, and 28, the disease incidence rate and indices of WL343HQ were lower than those of Dryland (*p* < 0.05). The net photosynthetic rate and stomatal conductance of the disease groups (WL + V and HD + V) were significantly lower than the control groups (WL and HD; Figures 1C,D; *p* < 0.05). Compared with the control, chlorophyll content of the disease groups (WL + V and HD + V) significantly decreased on days 7, 14, 21, and 28 (Figure 1E; *p* < 0.05). Chlorophyll content of WL343HQ inoculated with *V. alfalfae* (WL + V) was significantly higher than that of Dryland inoculated with *V. alfalfae* (HD + V; Figure 1E; *p* < 0.05). Compared with the control, MDA concentrations of HD + V group significantly increased by 38.87, 131.62, 162.24, and 174.57% on days 7, 14, 21, and 28, respectively (Figure 1F; *p* < 0.05). On days 14, 21, and 28, MDA concentration of WL + V group significantly increased by 98.48, 116.26, and 134.66% (Figure 1F; *p* < 0.05).

Inoculation of *V. alfalfae* significantly affected the SOD and POD activities of plants. Compared with the control, SOD activity of HD + V group increased by 44.08, 94.41, and 142.90% on days 7, 14, and 21, and decreased by 35.34% on day 28 (Figure 1G; *p* < 0.05). POD activity of HD + V group increased by 24.10 and 43.80% on days 7 and 14, and decreased by 28.56% on day 28 (Figure 1H; *p* < 0.05). SOD and POD activities of WL + V group increased by 61.67 and 31.36%, 103.24 and 62.70%, 115.56 and 44.79% on days 7, 14, and 21, respectively, and showed a decreasing trend on day 28 (Figures 1G,H; *p* < 0.05). SOD and POD activities of WL + V group were significantly higher than those of HD + V group. Compared with the control, JA concentrations of HD + V group significantly increased by 31.92, 65.66 and 47.69% on days 14, 21 and 28, respectively (Figure 1I; *p* < 0.05). The SA differences in HD + V group were not significant throughout the period of disease succession (Figure 1J). Compared with the control, the concentrations of JA and SA in WL + V group on days 7, 14, 21 and 28 significantly increased by 20.91 and 23.33%, 46.64 and 48.11%, 82.64 and 40.37%, 52.86 and 31.40%, respectively (Figures 1I,J; *p* < 0.05). Compared with the control, NO and H_2_O_2_ contents of HD + V group increased by 58.91 and 28.35%, 44.11 and 68.34%, 64.76 and 97.98% on days 14, 21 and 28, respectively (Figures 1K,L; *p* < 0.05). The NO and H_2_O_2_ contents of WL + V group significantly increased by 49.61 and 43.21%, 45.91 and 56.30%, 19.27 and 47.03%, 13.29 and 19.13% on days 7, 14, 21, and 28, respectively (Figures 1K,L; *p* < 0.05).

### Analysis of Differentially Expressed Genes in WL343HQ

**Figure 2:**
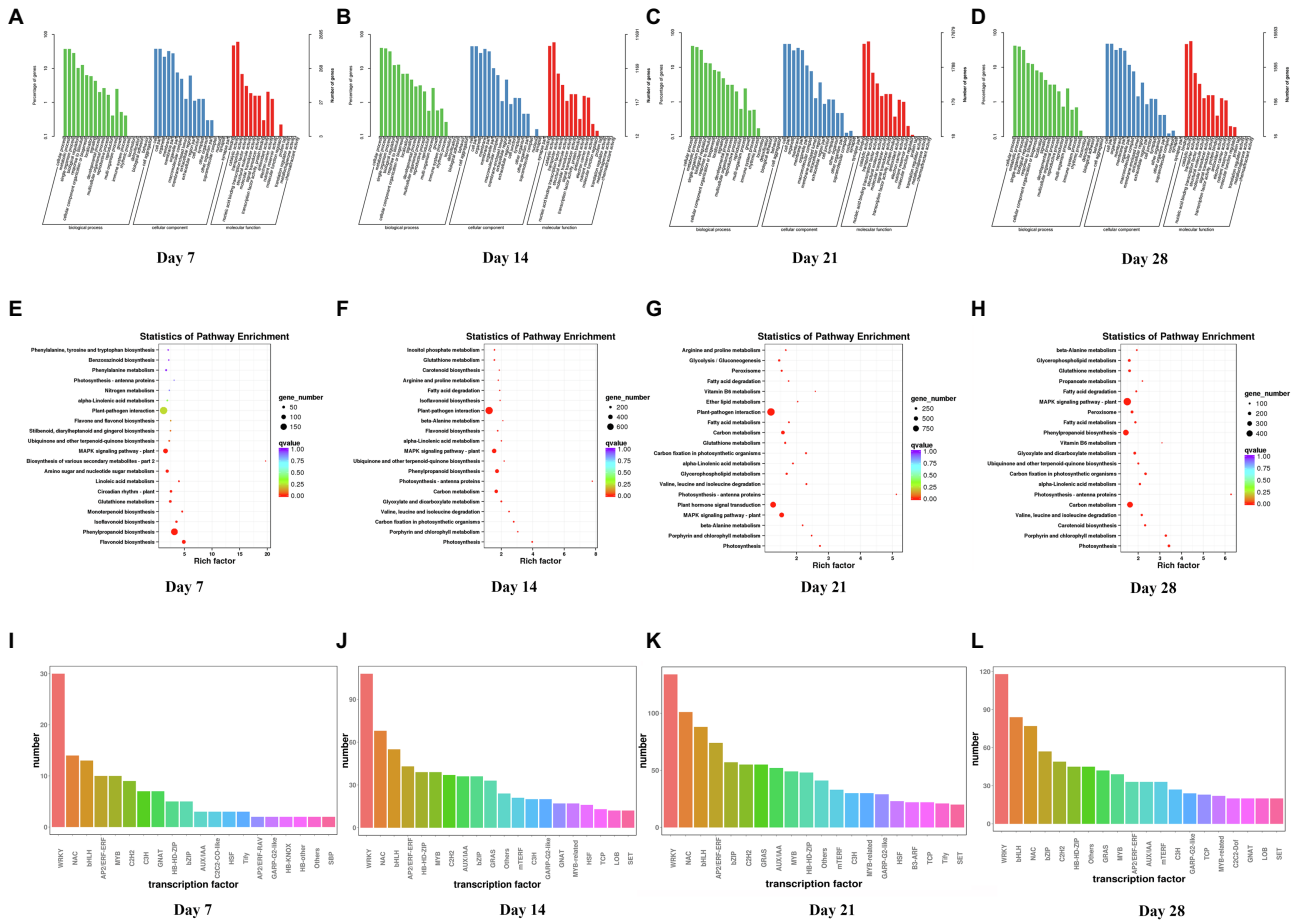
GO classification, KEGG enrichment analysis and transcription factor prediction of DEGs between WL343HQ inoculated with *Verticillium alfalfae* (WL + V) and control group (WL) on days 7, 14, 21, and 28. **(A–D)** GO classification of DEGs on days 7, 14, 21, and 28. **(E–H)** KEGG enrichment analysis of DEGs on days 7, 14, 21, and 28. **(I–L)** Transcription factor prediction of DEGs on days 7, 14, 21, and 28. *n* = 3 per group for each time point.

Compared with the control, the numbers of DEGs of WL343HQ inoculated with *V. alfalfae* (WL + V) on days 7, 14, 21 and 28 were 3,113, 13,526, 21,064, and 18,217, respectively. GO classification, KEGG enrichment analysis and transcription factor prediction were performed for DEGs. GO classification included 3 major categories, such as biological processes, cellular components and molecular functions (Figures 2A–D). KEGG enrichment analysis of DEGs on days 7, 14, 21, and 28 (Figures 2E–H) revealed that DEGs were mainly enriched in carbon fixation in photosynthetic organisms (ko00710), plant-pathogen interaction (ko04626), MAPK signaling pathway-plant (ko04016), glutathione metabolism (ko00480), photosynthesis-antenna proteins (ko00196) and α-Linolenic acid metabolism (ko00592). By transcription factor prediction, all DEGs were categorized into transcription factor families, and the top 20 transcription factor families with the highest abundance were selected (Figures 2I–L). It was found that DEGs of WL + V group on days 7, 14, 21, and 28 were mainly categorized into the following transcription factor families, including WRKY, bHLH, NAC, MYB, C2H2, HSF, bZIP, mTERF, AP2/ERF-ERF, C3H, TCP, GARP-G2-like, HB-HD-ZIP, and AUXIAA. The numbers of transcription factors were the highest in the WRKY, NAC and bHLH families. The numbers of transcription factors in WRKY family on days 7, 14, 21, and 28 were 30, 108, 134, and 118, respectively. The numbers of transcription factors in bHLH family were 13, 55, 88, and 84 on days 7, 14, 21, and 28. The numbers of transcription factors in NAC family were 14, 68, 101, and 77 on days 7, 14, 21, and 28. To investigate the specific changes in genes on days 7, 14, 21, and 28, venn diagrams were performed for the four groups, Wa vs. WVa, Wb vs. WVb, Wc vs. WVc, and Wd vs. WVd (Supplementary Figure 1A), the numbers of DEGs specific to the four differential groups were 673, 1954, 6,082 and 3,916, respectively. The number of DEGs co-expressed in the four differential groups was 1,539.

**Figure 3:**
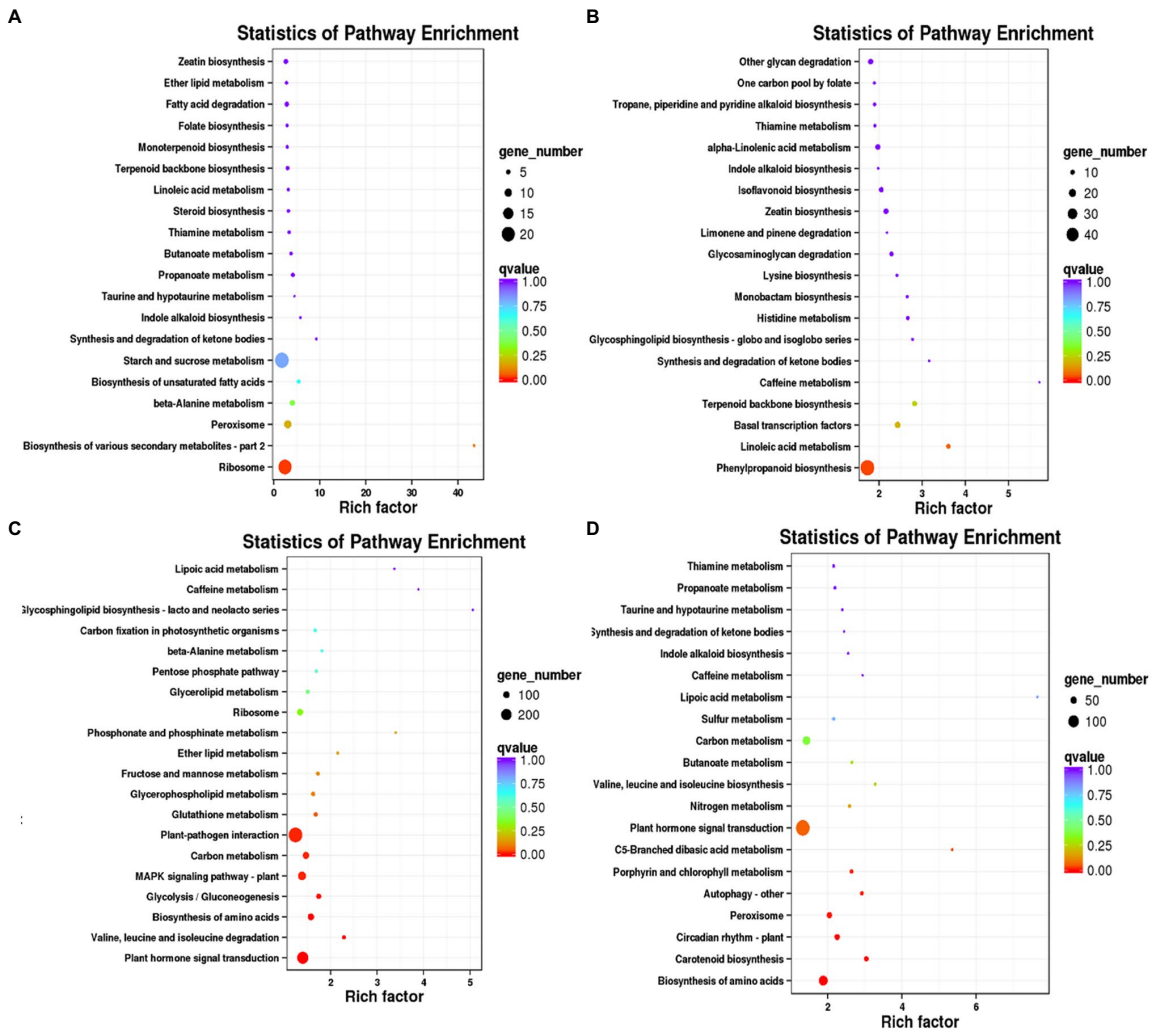
KEGG enrichment analysis of DEGs specific to days 7, 14, 21, and 28 in WL343HQ inoculated with *Verticillium alfalfae* (WL + V). **(A)** KEGG enrichment analysis of DEGs specific to days 7. **(B)** KEGG enrichment analysis of DEGs specific to days 14. **(C)** KEGG enrichment analysis of DEGs specific to days 21. **(D)** KEGG enrichment analysis of DEGs specific to days 28.

KEGG enrichment analysis were performed for DEGs specific to days 7, 14, 21, and 28, respectively. A 673 DEGs on day 7 were mainly enriched in ribosome (ko03010), biosynthesis of various secondary metabolites-part 2 (ko00998) and starch and sucrose metabolism (ko00500; Figure 3A). A 1954 DEGs on day 14 were predominantly enriched in phenylpropanoid biosynthesis (ko00940), linoleic acid metabolism (ko00591), basal transcription factors (ko03022) and caffeine metabolism (ko00232; Figure 3B). A 6,082 DEGs on day 21 were mainly enriched in plant hormone signal transduction (ko04075), biosynthesis of amino acids (ko01230), MAPK signaling pathway-plant (ko04016) and plant-pathogen interaction (ko04626; Figure 3C). A 3,916 DEGs on day 28 were mainly enriched in the biosynthesis of amino acids (ko01230), carotenoid biosynthesis (ko00906), peroxisome (ko04146) and plant hormone signal transduction (ko04075; Figure 3D).

### Analysis of Differentially Expressed Genes in Dryland

**Figure 4:**
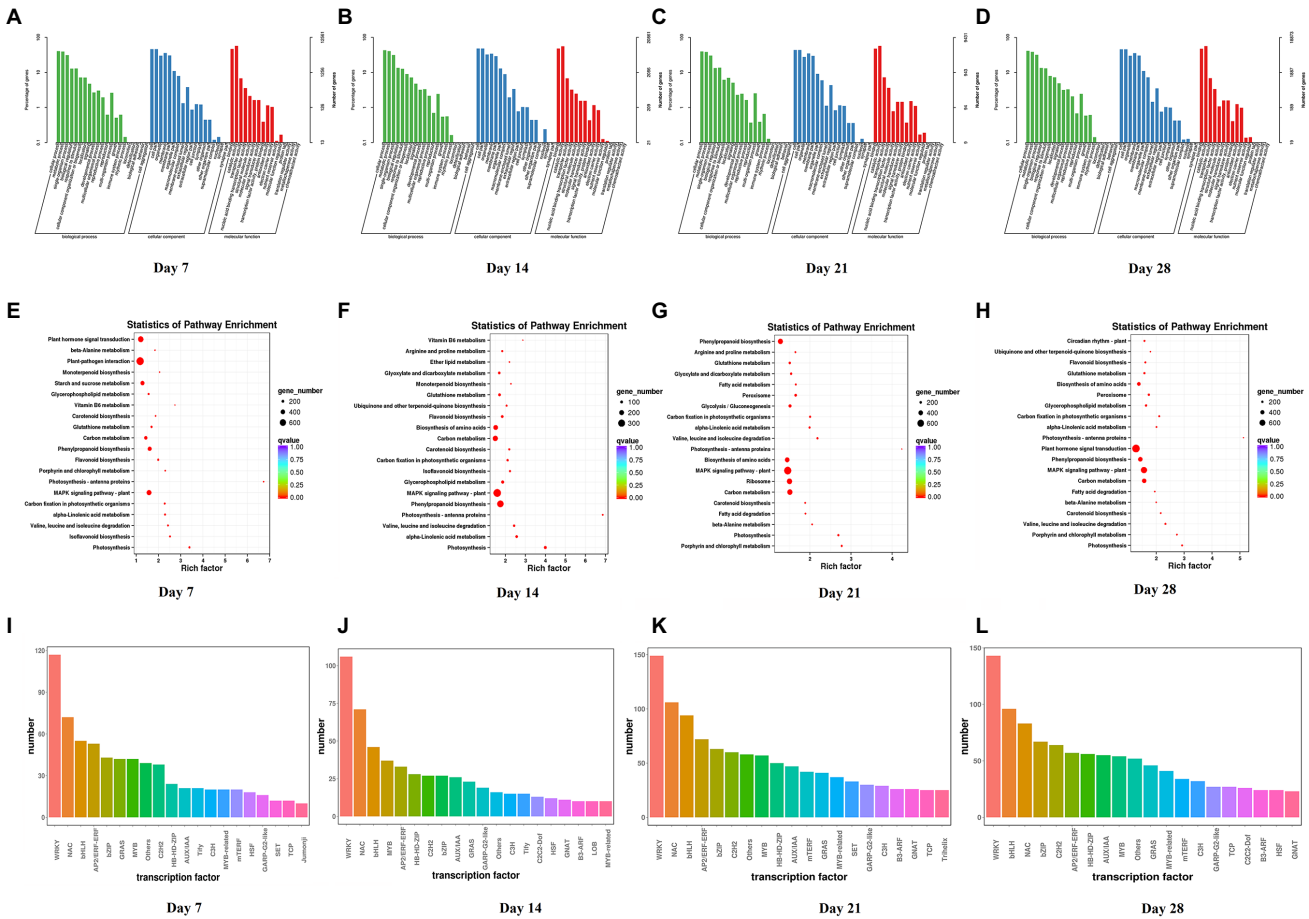
GO classification, KEGG enrichment analysis and transcription factor prediction of DEGs between Dryland inoculated with *Verticillium alfalfae* (HD + V) and control group (HD) on days 7, 14, 21, and 28. **(A–D)** GO classification of DEGs on days 7, 14, 21, and 28. **(E–H)** KEGG enrichment analysis of DEGs on days 7, 14, 21, and 28. **(I–L)** Transcription factor prediction of DEGs on days 7, 14, 21, and 28. *n* = 3 per group for each time point.

**Figure 5:**
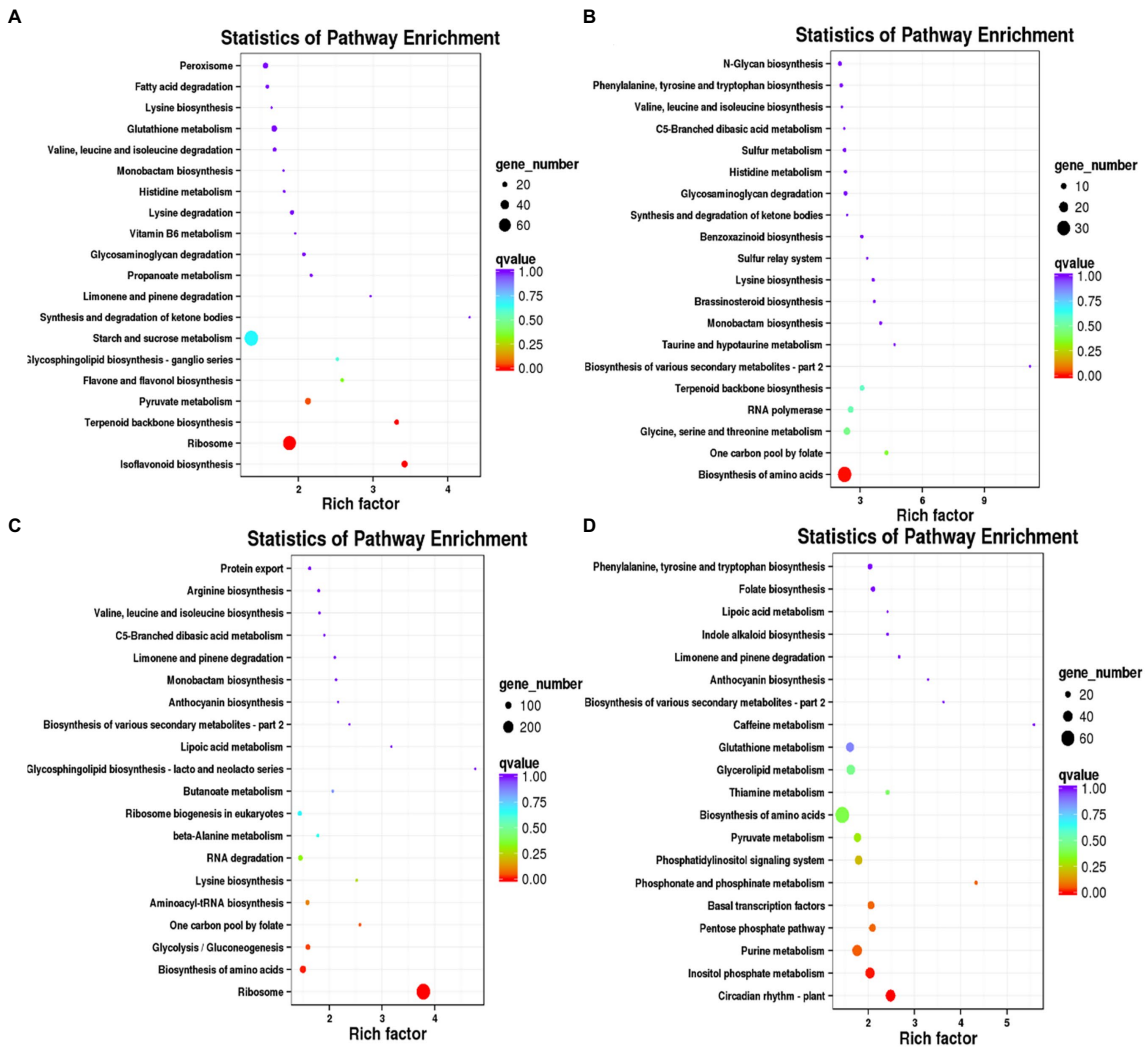
KEGG enrichment analysis of DEGs specific to days 7, 14, 21, and 28 in Dryland inoculated with *Verticillium alfalfae* (HD + V). **(A)** KEGG enrichment analysis of DEGs specific to days 7. **(B)** KEGG enrichment analysis of DEGs specific to days 14. **(C)** KEGG enrichment analysis of DEGs specific to days 21. **(D)** KEGG enrichment analysis of DEGs specific to days 28.

Compared with the control, the numbers of DEGs of Dryland inoculated with *V. alfalfae* (HD + V) on days 7, 14, 21, and 28 were 14,809, 11,127, 24,460, and 22,295, respectively. GO classification was also mainly in 3 major categories: biological processes, cellular components and molecular functions (Figures 4A–D). KEGG enrichment analysis of DEGs on days 7, 14, 21, and 28 (Figures 4E–H) revealed that DEGs were mainly enriched in valine, leucine and isoleucine degradation (ko00280), MAPK signaling pathway-plant (ko04016), photosynthesis (ko00195), photosynthesis-antenna proteins (ko00196), and α-Linolenic acid metabolism (ko00592). All DEGs were categorized into transcription factor families, and the top 20 transcription factor families with the highest abundance were selected (Figures 4I–L). Consistent with the results of WL343HQ (WL + V), DEGs of HD + V group on days 7, 14, 21, and 28 were mainly categorized into transcription factors of the WRKY, NAC and bHLH families. Venn diagrams were performed for the four groups, including Ha vs. HVa, Hb vs. HVb, Hc vs. HVc, and Hd vs. HVd (Supplementary Figure 1B), the numbers of DEGs specific to the four groups were 2,974, 1,437, 6,160, and 4,480, respectively. The number of DEGs co-expressed in the four groups was 5,277. KEGG enrichment analysis were performed for DEGs specific to days 7, 14, 21 and 28, respectively. A 2,974 DEGs on day 7 were mainly enriched in isoflavonoid biosynthesis (ko00943), ribosome (ko03010) and terpenoid backbone biosynthesis (ko00900; Figure 5A). A 1,437 DEGs on day 14 were mainly enriched in biosynthesis of amino acids (ko01230), glycine, serine and threonine metabolism (ko00260), RNA polymerase (ko03020) and terpenoid backbone biosynthesis (ko00900; Figure 5B). A 6,160 DEGs on day 21 were mainly enriched in ribosome (ko03010), biosynthesis of amino acids (ko01230) and aminoacyl-tRNA biosynthesis (ko00970; Figure 5C). A 4,480 DEGs on day 28 were mainly enriched in the circadian rhythm-plant (ko04712), inositol phosphate metabolism (ko00562), pentose phosphate pathway (ko00030) and basal transcription factors (ko03022; Figure 5D).

Furthermore, we compared the DEGs between the less resistant HD + V group and the highly resistant WL + V group. The number of DEGs on days 7, 14, 21, 28 were 5,432, 2,202, 2,705 and 1,259, respectively. GO classification and KEGG enrichment analysis were also performed for DEGs. GO classification included 3 major categories, such as biological processes, cellular components and molecular functions (Supplementary Figures 2A–D). KEGG enrichment analysis of DEGs on days 7, 14, 21 and 28 (Supplementary Figures 2E–H) revealed that DEGs were mainly enriched in photosynthesis (ko00195), photosynthesis-antenna proteins (ko00196), MAPK signaling pathway-plant (ko04016), carbon fixation in photosynthetic organisms (ko00710), starch and sucrose metabolism (ko00500) and carbon metabolism (ko01200). To investigate the specific changes in genes on days 7, 14, 21 and 28, venn diagrams were performed for the four groups, HVa vs. WVa, HVb vs. WVb, HVc vs. WVc, and HVd vs. WVd (Supplementary Figure 1C), the numbers of DEGs specific to the four differential groups were 4,050, 1,250, 1,695, and 772, respectively. The number of DEGs co-expressed in the four differential groups was 99.

### qPCR Validation of Differentially Expressed Genes

To verify the authenticity of the transcriptional data, 12 DEGs were randomly selected for qPCR validation. The results showed that the expressions of 12 DEGs were consistent with the trend of FPKM values of transcriptome sequencing, thus confirming the accuracy of transcriptome analysis (Supplementary Figure 3).

### Weighted Gene Co-expression Network Analysis

DEGs were screened and 17,414 genes were selected for WGCNA. When the average connectivity tended to 0, a scale-free network could be obtained with a threshold = 11 and *R*^2^ ≥ 0.85 (Supplementary Figure 4A,B). An adjacency matrix was constructed and converted into a topological overlap matrix to reflect the correlation strength in the co-expression network. The clustering of genes was achieved by the dissimilarity. The division of gene modules was realized by dynamic shearing algorithm, and the minimum number of genes in each module was set to 100, then 14 gene co-expression modules were initially obtained by calculating the feature vector of each module and merging similar modules (Supplementary Figure 4C). The module eigenvector values were calculated to obtain the dissimilarity between the modules, which was used to get the eigenvector gene proximity heatmap of each module (Supplementary Figure 4C). The relationships between the modules can be seen through the heatmap. Moreover, through the module clustering tree, the modules were further reduced in number compared to the pre-merger (Supplementary Figure 4E). After merging the modules, the distribution of gene expression in each module for each sample can be clearly displayed in the heatmap (Supplementary Figure 4F).

**Figure 6:**
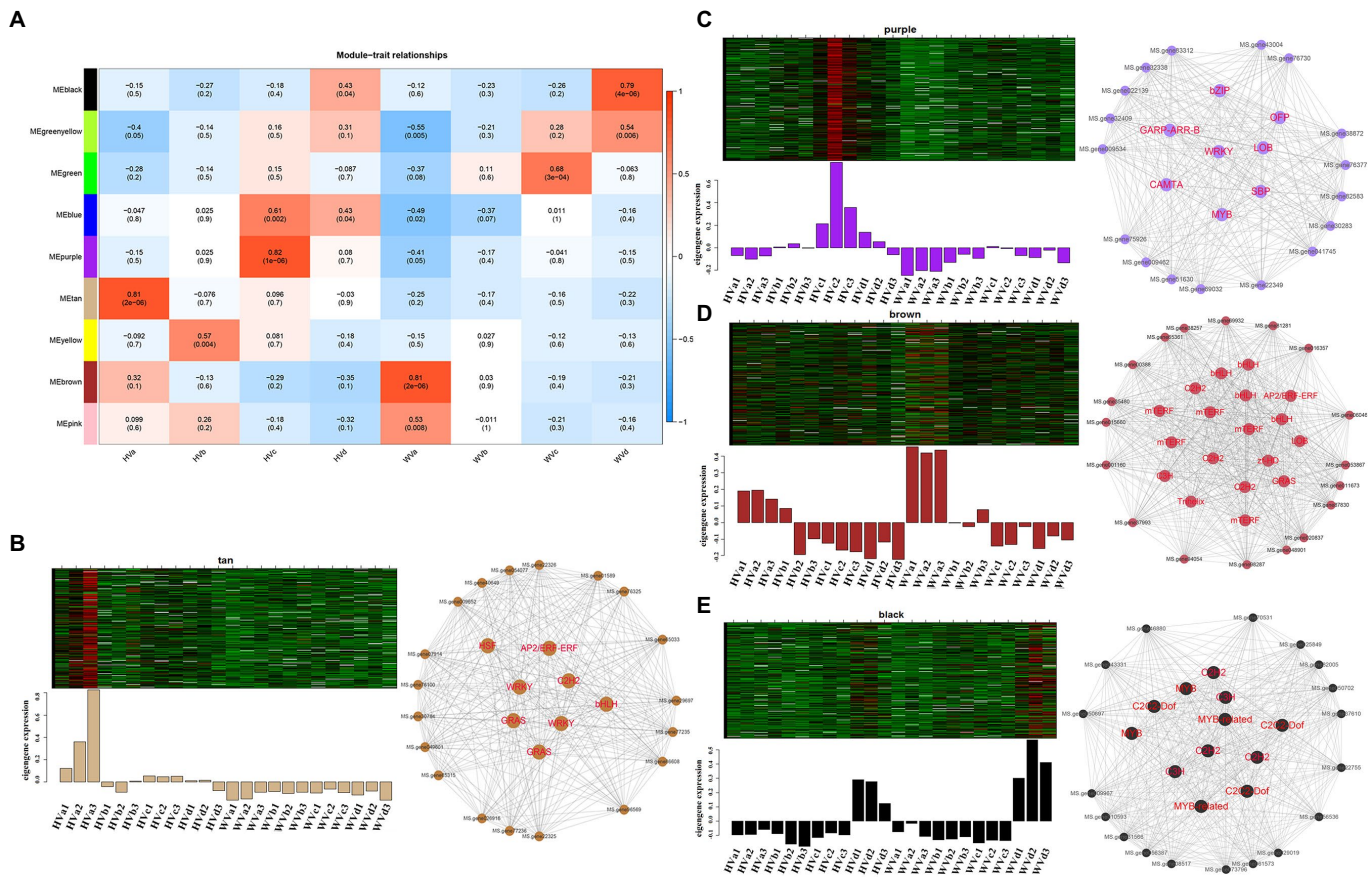
Weighted gene co-expression network analysis (WGCNA) of module eigengenes in corresponding modules. **(A)** Heatmap of correlation between treatment groups and corresponding modules. **(B)** Heatmap and barplots of module eigengenes expression, and visualization of core genes of tan module. **(C)** Heatmap and barplots of module eigengenes expression, and visualization of core genes of purple module. **(D)** Heatmap and barplots of module eigengenes expression, and visualization of core genes of brown module. **(E)** Heatmap and barplots of module eigengenes expression, and visualization of core genes of black module.

Among the final 9 gene co-expression modules, genes in four modules, tan, purple, brown and black, showed high correlation with HVa, HVc, WVa and WVd, respectively (Figure 6A). The first row in each box was the *R*-value and the second row was the *p*-value. *R*-value represented the correlation coefficient between the co-expression modules and the samples. We primarily screened the core genes of the four corresponding modules by intra-module significance value *R* and intramodular connectivity of the genes, and the screening condition was *R* ≥ 0.8 and intramodular connectivity ≥0.8. The total number of genes in the tan module corresponding to the HVa was 322, and the number of core genes in this module was 27. The heatmap of tan module genes expression and the barplots of module eigengenes expression values were shown in Figure 6B. The expression of module eigengenes in the three replicates of HVa treatment was higher than other treatments, and the visualization result of 27 core genes was shown in Figure 6B. The total numbers of genes in the purple, brown and black modules were 398, 2037 and 485, and the numbers of core genes were 25, 36 and 30, respectively (Figures 6C–E). In the purple, brown and black modules, the expressions of module eigengenes in the replicates of corresponding HVc, WVa and WVd treatments were higher than other treatments.

## Discussion

Pathogen infestation could reduce photosynthesis in plants. The reason is that pathogen infestation restrained acyclic photosynthetic phosphorylation with NADP or ferricyanide as the acceptor, ultimately inhibiting the photochemical reactions in photosynthesis (Labudda et al., 2020). In addition, pathogen infestation damaged pigment proteins involved in photosynthesis (Saleem et al., 2020) and caused chlorophyll breakdown and stomatal closure, blocking plant CO_2_ supply and thus limiting photosynthesis (Xia et al., 2016; Gao et al., 2018). It was reported that inoculation with *V. dahliae* decreased photosynthetic rate and stomatal conductance in pepper and reduced plant biomass and yield ultimately (Pascual et al., 2009). In this study, the net photosynthetic rate, stomatal conductance, and chlorophyll content of WL343HQ (WL + V) were higher than those of Dryland (HD + V), which was related to the lower disease incidence and disease index of WL343HQ (WL + V). In the photosynthesis-antenna proteins pathway (ko00196), *MS.gene64487*, *MS.gene018915*, *MS.gene031016* and *MS.gene031989*, which synthesize the light-harvesting chlorophyll protein complexes Lhca1-Lhca4, were downregulated in WL343HQ. Meanwhile, 12 DEGs, such as *MS.gene019953*, *MS.gene025268* and *MS.gene017490*, were downregulated in Dryland, thereby blocking Lhca1-Lhca7 synthesis and further restraining photosynthesis in Dryland. In addition, DEGs in Dryland on days 7, 14, 21, 28 were all enriched in photosynthesis (ko00195). In this pathway, *MS.gene038239*, *MS.gene47675*, *MS.gene47675* and *MS.gene024886*, which are involved in the synthesis of PsbP, PsbB, PsbW and Psb27 in photosystem II, were downregulated in expression. Downregulation of *MS.gene91444*, *MS.gene92305*, *MS.gene038025* and *MS.gene013347* inhibited the synthesis of the subunit proteins PsaD, PsaE, PsaF and PsaK, which are essential components of photosystem I and play important roles in the post-transcription of plastids (Rochaix et al., 2004). Compared with WL + V group, the above reasons led to lower photosynthesis and chlorophyll content of HD + V group.

MDA is the final decomposition product of membrane lipid peroxidation and its level can reflect the degree of stress and tissue peroxidative damage (Del Rio et al., 2005). In our study, the lower MDA content of WL + V group indicated that the membrane lipid peroxidation of HD + V group was stronger than that of WL + V group. In the plant-pathogen interaction pathway, upregulated DEGs in WL + V group were more than HD + V group and involved in the biosynthesis of calcium-dependent protein kinases, which were related to plant stress response and disease resistance (Ning et al., 2019). In HD + V group, the majority of DEGs involved in the isoflavone biosynthetic pathway were downregulated, which affected the biosynthesis of UDP-glycosyltransferase and cytochrome P450, thereby reducing the effective response to pathogen infection and resistance to stress of plants (Bolwell et al., 1994; Giovannetti et al., 2015). This is the reason for the lower MDA content in the WL + V group throughout the experiment. The enhanced SOD and POD enzyme activities inhibited the growth and sporulation of mycelium of pathogenic fungi and induced lignification of the host plant (Asthir et al., 2010). By scavenging reactive oxygen species, cell damage was reduced by SOD and POD enzymes (Wang et al., 2012). In the current study, the SOD and POD enzyme activities of HD + V group exhibited a trend of increasing and then decreasing on day 28. The reason for the fall on day 28 may be that the rapid accumulation of reactive oxygen species in the plant exceeded the threshold value, resulting in the disruption of the normal reactive oxygen scavenging enzyme system and reduction of SOD and POD enzyme activities. DEGs of WL + V and HD + V group on days 7, 14, 21 and 28 were mainly categorized into transcription factors of the WRKY, NAC and bHLH families, which can activate antioxidant enzymes, such as SOD and POD, for enhancing stress response and disease resistance of plants (Varaud et al., 2011; Ishihama and Yoshioka, 2012). Total and upregulated DEGs of the WRKY, NAC and bHLH families were more in WL + V group than in HD + V group, indicating that WL343HQ can produce more antioxidant enzymes against pathogens than Dryland, which was in agreement with our results. The numbers of expressed genes of WRKY, bHLH and NAC transcription factors in both WL + V and HD + V groups were gradually increased on days 7, 14 and 21, and decreased on day 28, indicating that the antioxidant system of plant was gradually retarded on day 28. In addition to WRKY, NAC and bHLH, transcription factor families such as HSF, MYB, SBP, mTERF, zf-HD, C2C2-CO-like, LIM, Trihelix and PLATZ were also involved in plant disease resistance, which was consistent with previous studies (Du et al., 2009a; Scharf et al., 2012).

H_2_O_2_, NO, JA and SA are common signaling substances that can activate the expression of plant disease-resistant defense genes and induce the defensive responses synergistically or individually (Delledonne et al., 1998; Durner et al., 1998). In addition, H_2_O_2_ and NO are strongly oxidative and excessive accumulation can cause cell damage. In this study, the H_2_O_2_ content of WL + V group was significantly higher than that of HD + V group on days 7 and 14. This was related to the role of H_2_O_2_ as signaling substance against pathogen invasion and mycelium expansion (Zhang et al., 1995; Dat et al., 2000; Mellersh et al., 2002). On day 7 and 14, the majority of DEGs in WL + V group were upregulated in the basal transcription factor pathway and involved in the biosynthesis of receptor-like protein kinase, which was mainly correlated to signal reception and transduction processes in plants (Sakamoto et al., 2019), resulting in higher levels of the signal substances H_2_O_2_ and NO in WL + V group on days 7 and 14. The following decrease on the days 21 and 24 was mainly due to higher SOD and POD enzyme activities. In the glutathione pathway, there were more DEGs in WL + V group. Among them, *MS.gene019928*, *MS.gene65554*, *MS.gene74672* and *MS.gene063650* involved in glutathione reductase and glutamate-cysteine ligase synthesis were upregulated in expression. These enzymes can assist SOD and POD in scavenging non-radical reactive oxygen species H_2_O_2_ in plants (Gill and Tuteja, 2010), which was the reason for lower H_2_O_2_ in WL + V group. It was reported that infection with *Verticillium longifolium* increased JA content in rape (Ratzinger et al., 2009). The changes of JA and SA concentrations indicated that WL + V group induced JA and SA signaling pathways, while HD + V group only induced JA signaling pathway. DEGs in WL343HQ on days 7, 14, 21, 28 were all enriched in α-linolenic acid metabolism (ko00592). Among them, the upregulated expression of *MS.gene07270*, *MS.gene20792* and *MS.gene20842* promoted the synthesis of triacylglycerol lipase, thus increasing the content of α-linolenic acid. JA was formed by the oxidation process of α-linolenic acid in the chloroplast membrane (Wasternack and Song, 2017). In addition, upregulated *MS.gene031651* was involved in the synthesis of acyl-CoA oxidase, which promoted the synthesis of JA-CoA, an important precursor substance for the synthesis of JA. Compared with HD + V group, the above reasons led to higher JA concentrations in WL + V group. DEGs in WL343HQ on days 7, 14, 21, 28 were all enriched in the phenylpropanoid biosynthesis (ko00940). Phenylalanine ammonia-lyase-mediated phenylalanine pathway is the most important pathway for SA biosynthesis (An and Mou, 2011) and WL + V group had more upregulated genes in this pathway than HD + V group, such as *MS.gene003278*, *MS.gene006350*, *MS.gene062216*, *MS.gene34438* and *MS.gene44482*, resulting in higher SA concentrations in WL + V group.

In addition to the above-mentioned physiological and biochemical changes, alfalfa also resists Verticillium wilt through other mechanisms. In WL + V group, the DEGs specific to day 7, 14, 21 and 28 were mostly upregulated and involved in plant disease defense processes by participating in the biosynthesis of isoflavone reductase homolog in the secondary metabolite biosynthesis pathway (Tee, 2009). In the starch and sucrose metabolism, genes were involved in the biosynthesis of β-1,3 glucanase, which was an enzyme that induced plant resistance to pathogenic invasion and inhibited the growth of pathogenic fungi in plants (Thakur, 2021). In the MAPK signaling pathway-plant, the special DEGs on day 21 were mostly upregulated and associated with the biosynthesis of calmodulin, the most important receptor for Ca^2+^, which regulated the biosynthesis of SA and thus affected the defense and resistance processes of plants against pathogenic fungi (Du et al., 2009b). In the amino acid biosynthetic pathway, the special DEGs on day 28 were involved in the synthesis of zinc finger proteins, which was associated with plant growth and resistance mechanisms in various biotic and abiotic stresses (Feurtado et al., 2011; Giri et al., 2011). As for the HD + V group, most of the special DEGs were downregulated compared with the control, which led to the reduction of disease resistance in Dryland. The special DEGs on day 14 were mostly downregulated in HD + V group and mainly involved in the biosynthesis of serine acetyltransferase and cysteine synthase, both of which were associated with the biosynthesis of cysteine. Cysteine was important in reversible oxidative modifications, plant development regulation and stress response in plants (Richau et al., 2012). Among the special DEGs on day 21, the downregulated genes were involved in the synthesis of heat shock 70 kDa protein, which protected cells from damage caused by various stressors (Gomez-Pastor et al., 2018). The above results indicated that WL + V group was more resistant to Verticillium wilt compared with HD + V group.

## Conclusion

Inoculation with *V. alfalfae* significantly affected net photosynthetic rate, stomatal conductance, chlorophyll content, MDA content, JA and SA concentrations, NO and H_2_O_2_ contents both in WL343HQ and Dryland inoculated with *Verticillium alfalfae*. SOD and POD enzyme activities and JA concentrations of both two disease varieties showed a trend of first increasing and then decreasing. The net photosynthetic rate, stomatal conductance, SOD and POD enzyme activities, and JA and SA concentrations of WL + V group were higher than those of HD + V group, suggesting that the defensive responses of WL + V group were stronger than HD + V group. The number of DEGs between HD + V group and WL + V group on days 7, 14, 21 and 28 were 14,809 and 3,113, 11,127, and 13,526, 24,460 and 21,064, 22,295 and 18,217, respectively. According to KEGG enrichment analysis, most of DEGs were found to be enriched in photosynthesis, photosynthesis-antenna proteins, linoleic acid metabolism, carbon fixation in photosynthetic organisms, MAPK signaling pathway-plant, phenylpropanoid biosynthesis, and glutathione metabolism pathways. Transcription factor prediction revealed a high number of transcription factors in the WRKY, NAC, and bHLH families. WGCNA analysis showed that the number of transcription factors related to plant growth and disease resistance was higher in the corresponding modules of WVa and WVd than in the corresponding modules of HVa and HVc. The number of core genes of the four corresponding modules including HVa, HVc, WVa, and WVd was 27, 25, 36 and 30, respectively. This work provides new insights into the change by which *V. alfalfae* affect physiological indicators and transcriptome at different time after inoculation. In the future, more in-depth studies are required to reveal the molecular mechanisms of the correlation between DEGs and physiological indicators of alfalfa.

## Data Availability Statement

The datasets presented in this study can be found in online repositories. The names of the repository/repositories and accession number(s) can be found at: https://www.ncbi.nlm.nih.gov/, PRJNA838389.

## Author Contributions

YL: project administration, supervision, experimental design, and funding acquisition. ZS: supervision, funding acquisition, validation, visualization, and writing—review and editing. FL: investigation, resources, validation, and data curation. XC: validation, data curation, and writing—original draft. BY: investigation and resources. YG and DW: methodology and visualization. All authors contributed to the article and approved the submitted version.

## Funding

This study was financially supported by National Nature Science Foundation of China (32061123004), Gansu Provincial Science and Technology Major Projects (No. 19ZD2NA002), and China Post-doctoral Science Foundation (2019M662893).

## Conflict of Interest

The authors declare that the research was conducted in the absence of any commercial or financial relationships that could be construed as a potential conflict of interest.

## Publisher’s Note

All claims expressed in this article are solely those of the authors and do not necessarily represent those of their affiliated organizations, or those of the publisher, the editors and the reviewers. Any product that may be evaluated in this article, or claim that may be made by its manufacturer, is not guaranteed or endorsed by the publisher.
