# Supplementary material for: Transcriptome Analysis Revealed the Molecular Response Mechanism of High-Resistant and Low-Resistant Alfalfa Varieties to Verticillium Wilt
Source: Front Plant Sci. 2022 Jun 16;13:931001. doi: 10.3389/fpls.2022.931001 (PMC9243768; doi:10.3389/fpls.2022.931001)
Supplement: Supplementary file 1 [file Data_Sheet_1.docx]

**Transcriptome Analysis Revealed the Molecular Response Mechanism of High-resistant and Low-resistant Alfalfa Varieties to Verticillium wilt**

**Fang Li^✝,1^, Xi Chen^✝,1^, Bo Yang^1^, Yingjie Guang^1^, Dandan Wu^1^, Zunji Shi^*,1^, and Yanzhong Li^*,1^**

^1^State Key Laboratory of Grassland Agro-ecosystems, Center for Grassland Microbiome, Gansu Tech Innovation Center of Western China Grassland Industry, College of Pastoral Agriculture Science and Technology, Lanzhou University, Lanzhou, 730000, China

**^✝^** These authors have contributed equally to this work and share first authorship

*** Correspondence:**  Dr. Zunji Shi and Dr. Yanzhong Li

Email: [shizj@lzu.edu.cn](mailto:shizj@lzu.edu.cn); liyzh@lzu.edu.cn

**KEYWORDS: alfalfa; Verticillium wilt; *Verticillium alfalfae*; resistance mechanism; transcriptomics**

**Supplementary Table 1** Gene information of q-PCR validation.

| Genes | Upstream primers | Downstream primers |
| --- | --- | --- |
| *MS.gene031684* | GATTGGATTTGGGAGTGGAT | TAATACACTGACAGGGATCC |
| *MS.gene03228* | TATCTAGCACTGATTTGCAT | AACCGAATCATACTTCTACA |
| *MS.gene021998* | CGCTTCCTTCATCTGTACAG | CGAATAACATCACCTTCCCA |
| *MS.gene72897* | GACAAGCTAACATGGTTACT | ACAAGAATATGAAGCTATAT |
| *MS.gene003278* | TAGTCTCATAGCAACAGCTC | CCTTCCATTGATTCTGTAGA |
| *MS.gene063650* | CACTTCCCACTCTTGCTCTA | CCAGCACAGTCAATGTGTTT |
| *MS.gene031651* | TCTGGGAACCAAGCGTCACC | TGCTGCATCAGAACATTGTT |
| *MS.gene014928* | TCACTCTCTGCGAAGTCTGC | AAATGGAGTCAACTGCATCA |
| *MS.gene019928* | ACGGTGAACCTGATTTGAAC | TTGCTCCTGTCTTATCAACC |
| *MS.gene07270* | TAATGTTAAGGTAAGAGCTG | AAGGAACGATCTCTCCACTT |
| *MS.gene64487* | TTCCAAACAGAGAAATATGT | GATCAGCAAGATGAGCCATC |
| *MS.gene06319* | GGGTTCTCCCTTATGTAACT | CGAATTAGATCCTTGGCACT |


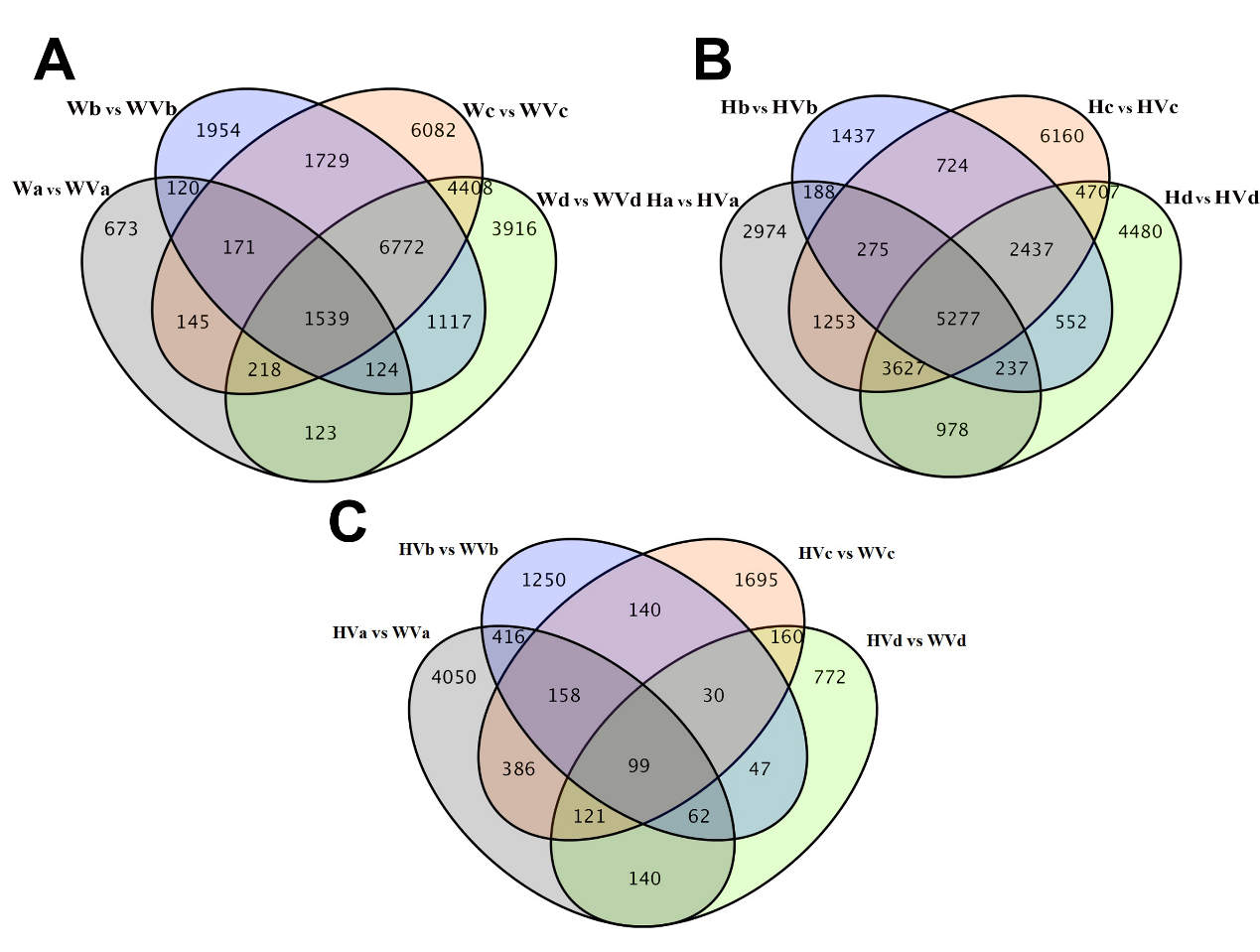


**Supplementary Figure 1** Venn diagrams of DEGs. (A) WL343HQ inoculated with *Verticillium alfalfae* (WL+V) and control groups (WL) on days 7, 14, 21 and 28. (B) Dryland inoculated with *Verticillium alfalfae* (HD+V) and control groups (HD) on days 7, 14, 21 and 28. (C) Dryland inoculated with *Verticillium alfalfae* (HD+V) group and WL343HQ inoculated with *Verticillium alfalfae* (WL+V) group on days 7, 14, 21 and 28. Data are presented as mean ± SD; n = 3 per group.


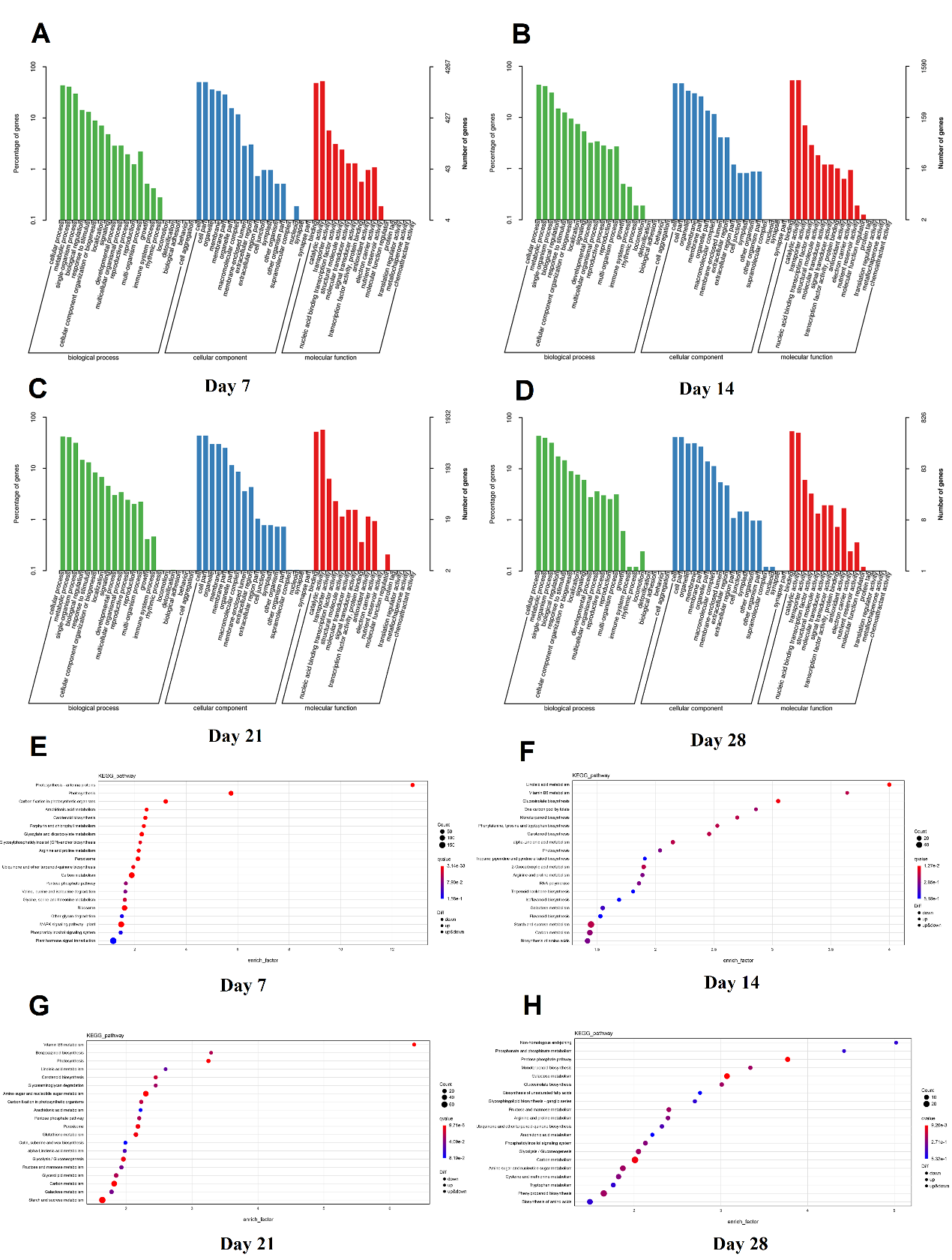


**Supplementary Figure 2** GO classification and KEGG enrichment analysis of DEGs between Dryland inoculated with *Verticillium alfalfae* (HD+V) and WL343HQ inoculated with *Verticillium alfalfae* (WL+V) on days 7, 14, 21 and 28. (A-D) GO classification of DEGs on days 7, 14, 21 and 28. (E-H) KEGG enrichment analysis of DEGs on days 7, 14, 21 and 28. n = 3 per group for each time point.


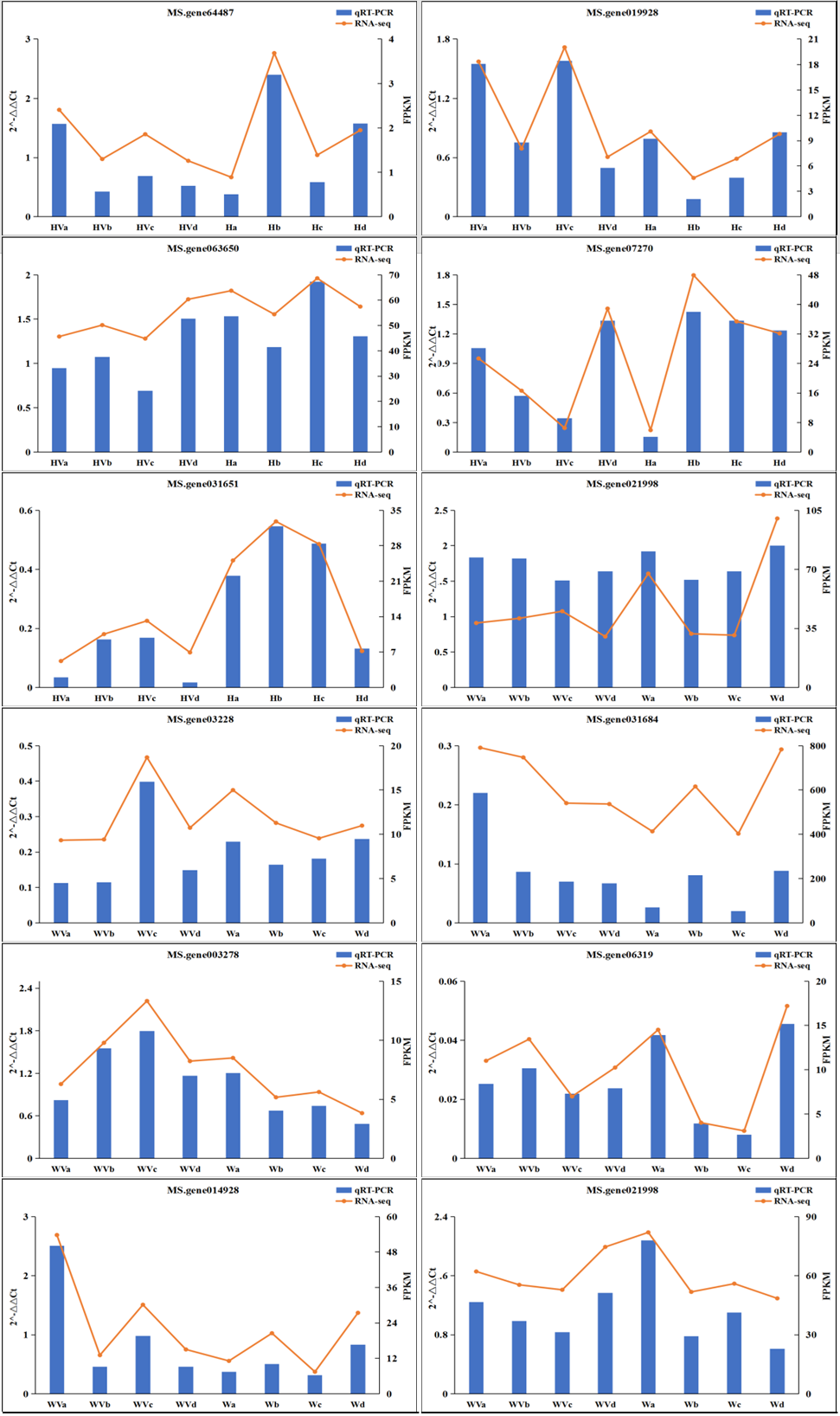


**Supplementary Figure 3** Barplots of qPCR validation of 12 randomly selected DEGs and the corresponding linear trend chart of FPKM values of transcriptome sequencing. FPKM, fragments per kilobase of transcript per million mapped reads.


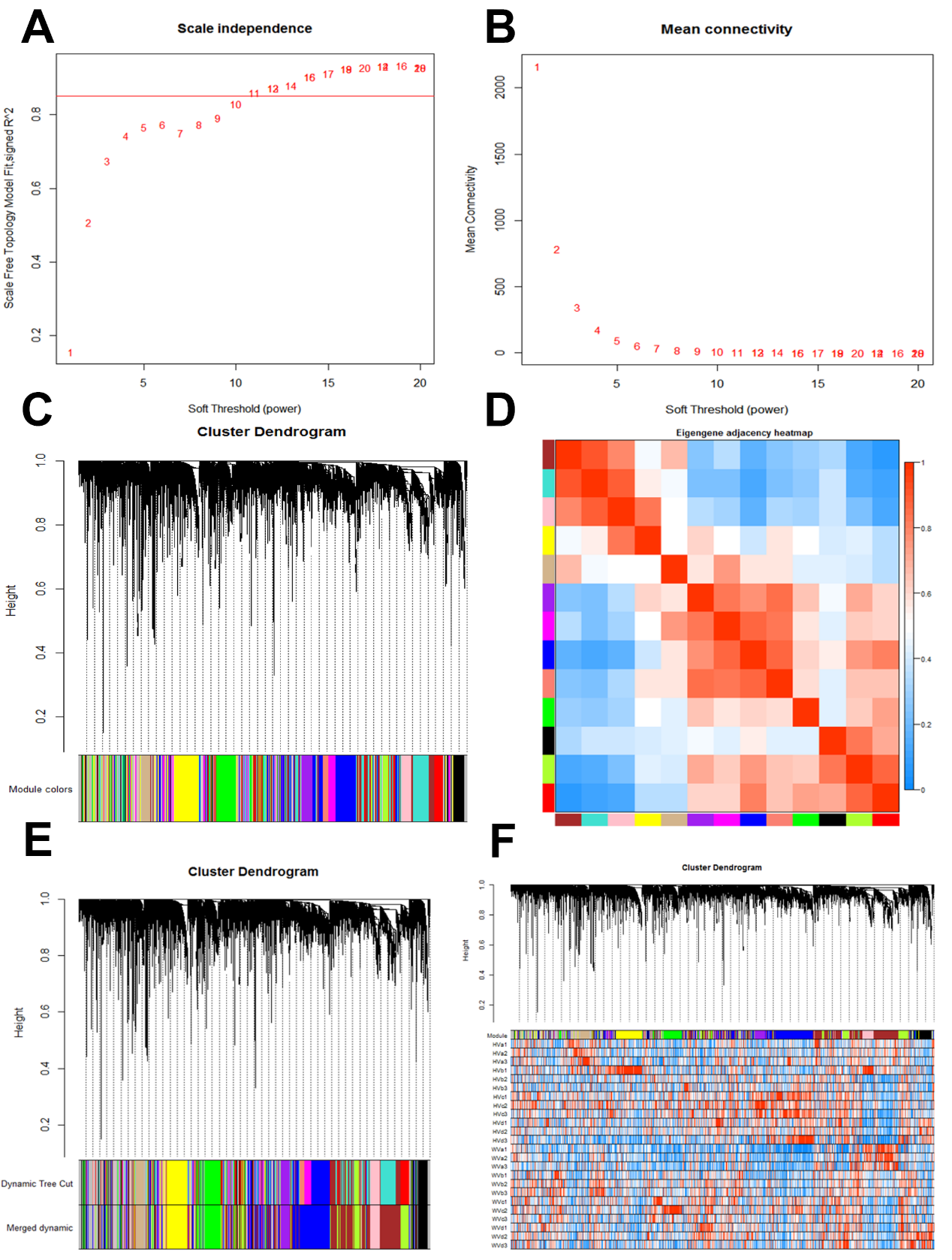


**Supplementary Figure 4** Gene clustering and modules merging of weighted gene co-expression network analysis (WGCNA). (A) The scale-free topology model fit R2. (B) Mean connectivity under different thresholds. (C) Gene clustering tree and initial gene co-expression modules. (D) Module eigengene adjacency heatmap between initial gene co-expression modules. (E) Gene clustering tree, and the modules before merging and after merging. (F) Gene clustering tree and heatmap of distribution of gene expression between each module and each sample.
